# Automatic and Fast Recognition of On-Road High-Emitting Vehicles Using an Optical Remote Sensing System

**DOI:** 10.3390/s19163540

**Published:** 2019-08-13

**Authors:** Hao Xie, Yujun Zhang, Ying He, Kun You, Boqiang Fan, Dongqi Yu, Mengqi Li

**Affiliations:** 1 Key Laboratory of Environmental Optics & Technology, Anhui Institute of Optics and Fine Mechanics, Chinese Academy of Sciences, Hefei 230031, China; 2 University of Science and Technology of China, Hefei 230026, China

**Keywords:** optical remote sensing system, emission data analysis, self-adaptive clustering database, automatic high-emitting recognition

## Abstract

Optical remote sensing systems (RSSs) for monitoring vehicle emissions can be installed on any road and provide non-contact on-road measurements, that allow law enforcement departments to monitor emissions of a large number of on-road vehicles. Although many studies in different research fields have been performed using RSSs, there has been little research on the automatic recognition of on-road high-emitting vehicles. In general, high-emitting vehicles and low-emitting vehicles are classified by fixed emission concentration cut-points, that lack a strict scientific basis, and the actual cut-points are sensitive to environmental factors, such as wind speed and direction, outdoor temperature, relative humidity, atmospheric pressure, and so on. Besides this issue, single instantaneous monitoring results from RSSs are easily affected by systematic and random errors, leading to unreliable results. This paper proposes a method to solve the above problems. The automatic and fast-recognition method for on-road high-emitting vehicles (AFR-OHV) is the first application of machine learning, combined with big data analysis for remote sensing monitoring of on-road high-emitting vehicles. The method constructs adaptively updates a clustering database using real-time collections of emission datasets from an RSS. Then, new vehicles, that pass through the RSS, are recognized rapidly by the nearest neighbor classifier, which is guided by a real-time updated clustering database. Experimental results, based on real data, including the Davies-Bouldin Index (DBI) and Dunn Validity Index (DVI), show that AFR-OHV provides faster convergence speed and better performance. Furthermore, it is not easily disturbed by outliers. Our classifier obtains high scores for Precision (PRE), Recall (REC), the Receiver Operator Characteristic (ROC), and the Area Under the Curve (AUC). The rates of different classifications of excessive emissions and self-adaptive cut-points are calculated automatically in order to provide references for law enforcement departments to establish evaluation criterion for on-road high-emitting vehicles, detected by the RSS.

## 1. Introduction

Vehicle emission are a major factor in urban air pollution, and car ownership continuously increases every year [1]. Thus, it is essential that we use available measures to monitor and control vehicle emissions. Generally, these measures consist of chassis and engine dynamometer tests, road-tunnel measurements, portable emission measurement systems (PEMS), plume chasing measurements, and optical remote sensing systems (RSSs). Chassis and engine dynamometer testing cannot reflect the real emission levels in on-road driving conditions [2], and road-tunnel methods are subject to geographical and environmental conditions [3]. PEMS and plume chasing measurement can precisely determine vehicle emissions, but PEMS take considerable time to install and uninstall these systems to transfer them between vehicles, and plume chasing measurements limit the speed and minimum distance for safety; these approaches are not suitable for monitoring a large number of vehicles. Further, their high price must be taken into consideration [4,5]. RSSs adopt non-dispersive infrared technology to detect CO, CO_2_, HC, and they use middle-infrared laser spectrum technology to detect NO; thus, RSSs can be used to perform non-contact on-road measurements [6]. An RSS can be installed on any road, rendering it a feasible and real-time measurement system for law enforcement departments to detect on-road high-emitting vehicles, where it is not viable to use the other three methods.

Many researchers have conducted studies with RSSs. Stedman and Bishop, who invented and developed it for a series of studies, were the pioneers of the RSS [7]. Kang et al. proposed a two-step location strategy using both, depth-first searching and greedy strategy, to find the minimum set of roads with traffic emission monitors, based on the digraph modeled from the traffic network [8]. Huang et al. researched the mechanism, applications, as well as a case study of RSS from Hong Kong. Their studies showed that the accuracy and number of vehicles affected by remote sensing screening programs were highly dependent on the cut-points, and that using fixed conservative cut-points in absolute concentrations (% or ppm) may be inappropriate [9]. Bernard et al. carried out a lot of research on RSS in Europe, and they used a laboratory limit to distinguish high-emitting vehicles [10,11]. Zhang et al. used a long short-term memory (LSTM) network to forecast vehicle emissions using multi-day observations by an RSS [12]. Even though many studies have been performed in different research fields using RSSs [13], little research has been carried out to automatically detect on-road high-emitting vehicles using this technology.

Usually, high-emitting vehicles and low-emitting vehicles are classified by the fixed cut-off concentrations of CO,HC, and NO. However, the set values for these cut-points lack a scientific basis [14]. RSS measurements are highly sensitive to multiple environmental factors, such as geographical conditions, meteorological conditions, air quality, wind, humidity, temperature, and so on, so the cut-off points between high-emitting and low-emitting vehicles are variable among different sites, times, and RSS equipment. To solve the above problem, we propose a novel adaptive method in this paper to establish cut-points and recognize high-emitting vehicles quickly and automatically. The system combines data analysis with clustering and classification methods from machine learning, and attempt to apply these methods to remote sensing monitoring of on-road high-emitting vehicles.

Firstly, 192,097 vehicle emission datasets, comprising CO,HC, and NO concentrations were collected by RSSs for 8 days. Secondly, we used three-dimensional and histogram statistics to analyze emission relationships. Secondly, an adaptive clustering algorithm was developed to rapidly label and rapidly divide the most recent 10,000 emission datasets into different high-emitting or low-emitting zones. Finally, new vehicles passing through the RSS were automatically and quickly classified into the corresponding zone, using a cluster database and nearest-neighbor classifier.

The core idea of our proposed algorithm is adaptive clustering. In general, there are five types of clustering methods in unsupervised learning: hierarchical-based clustering, density-based clustering, grid-based clustering, model-based clustering, and partition-based clustering. Hierarchical-based clustering generally includes Balanced Iterative Reducing and Clustering using Hierarchies (BIRCH) [15], Clustering Using REpresentatives (CURE) [16], RObust Clustering using linKs (ROCK) [17], and Chameleon [18]. The datasets are aggregated (bottom-up) or divided (up-bottom) into a series of nested subsets to form a tree structure. The hierarchical method has two major drawbacks; one is its high time-complexity. The second is that, once a mistake is made in one step, all subsequent steps will fail because of the inner greedy algorithm. Density-based clustering, which includes Density-Based Spatial Clustering of Applications with Noise (DBSCAN) [19], Ordering Points To Identify the Clustering Structure (OPTICS) [20], Distribution-Based Clustering of Large Spatial Databases (DBCLASD) [21], and DENsity-based CLUstEring (DENCLUE) [22], can divide datasets into arbitrary shapes by their regions of density, connectivity, and boundary, but it is extremely sensitive to the two initial parameters. Grid-based clustering divides the data space into grids and computes the density of each grid in order to identify high-density grids, and then adjacent high-density grids are integrated to become a cluster. Wave-Cluster [23] and STtatistical INfromation Grid (STING) [24] are typical examples of this clustering method. Model-based clustering optimizes the fit between the given data and the assumed model, which is based on statistics or neural network. The Gaussian Mixture Model (GMM) [25] and Self-Organizing Maps (SOM) [26] are representative of these two types of models. Partition-based clustering iteratively relocates datasets with a heuristic algorithm until optimization is achieved. There are many partitioning algorithms, such as K-Means, K-Means++ [27], kernel K-Means [28], K-Medoids [29], K-Modes [30], and Fuzzy C-means (FCM) [31]. K-Means++ and K-Medoids are used to restrain the sensitivity of the initial K values and outliers. K-modes and kernel K-means can be used in categorical or non-convex data, which traditional K-means are unable to do. FCM is a soft-threshold clustering method, compared with the hard-threshold of K-means.

The RSS in this study includes, fast and real-time features, as well as a large number of measured concentrations. Given the above advantages and disadvantages of the methods, our proposed approach applies a partition-based method. The most typical partition-based method, called K-means, is efficient for large datasets and has low time and space demands. However, K-means is sensitive to outliers and the selection of the initial K values. The adaptive method, called AFR-OHV was proposed in this paper to solve these two problems.

The remaining content in this paper is organized as follows. In Section 2, the emission datasets of the RSS are analyzed and our proposed method is introduced in detail. The experimental results and discussion are provided in Section 3. The paper is concluded in the last section.

## 2. Preliminaries

### 2.1. Emission Data Collection

**Figure 1:**
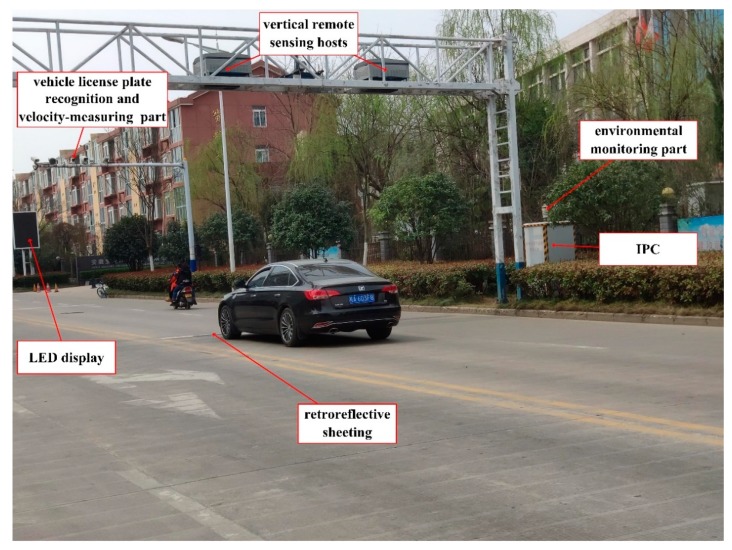
The optical remote sensing system for detecting on-road high-emitting vehicles.

The emission data were collected by an Optical Remote Sensing System (RSS) for 8 days from December 2018 to January 2019 on Xueyuan Road, Shijiazhuang City, Hopei Prov, China, Yangqiao Road, Hefei City, Anhui Prov, China, and Xincun Road, Zibo City, Shandong Prov, China respectively. The Optical Remote Sensing System, shown in Figure 1, consists of vertical remote sensing hosts, a velocity-measuring part, a vehicle license plate recognition part, an environmental monitoring part, an industrial personal computer (IPC), an LED display, and retroreflective sheeting. The advantage of a vertical RSS, compared with a road-side RSS, is that the monitoring of vehicles in a single lane is not disturbed by other vehicles simultaneously passing through other lanes, which can block the measurement light path when using road-side RSSs. Non-dispersion infrared technology is used to detect the concentration of $CO,CO_{2},HC,$ and middle-infrared laser spectrum technology is used to detect the concentration of NO by a vertical remote sensing host. When a vehicle passes through the vertical remote sensing host, the concentration of each emission gas in the exhaust plume is measured by the attenuation of light intensity, as defined by the Beer-Lambert Law [32],

(1) $$I_{\left(\lambda \right)}=I_{0(\lambda )}\exp(-\delta cL)$$

where $I_{0(\lambda )}$ and $I_{\left(\lambda \right)}$ are the initial and received light intensity, δ is the molecular absorption coefficient, c is the concentration of a particular gas, L is the absorption beam path and λ is the wavelength.

In the velocity-measuring part, the radar and laser detection technology measure the vehicle speed, and acceleration, respectively. A camera, a video capture card, and license plate automatic recognition software are integrated into the vehicle license plate recognition part. The temperature, relative humidity, wind speed, wind direction, atmospheric pressure, and gradient are obtained by the environmental monitoring part. The data collected by all sensors are uploaded to an industrial personal computer (IPC) for processing, so that on-road high-emitting vehicles can be recognized automatically. In addition, the license plate number, vehicle speed, and emission detection results are shown by the LED display in real time.

### 2.2. Collected Data Analysis

**Figure 2:**
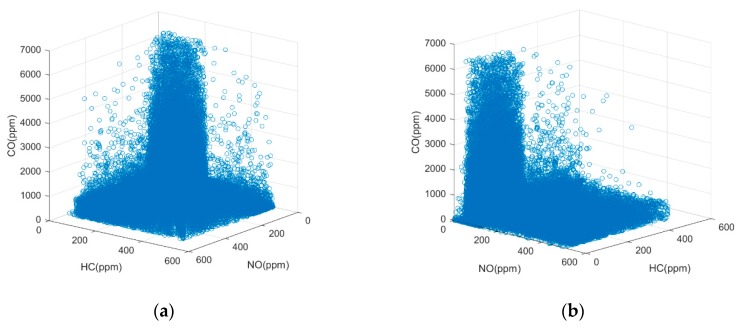
The concentration distribution of three main types of emissions collected in 192,097 datasets: (**a**) 3D front view; (**b**) 3D side view.

**Figure 3:**
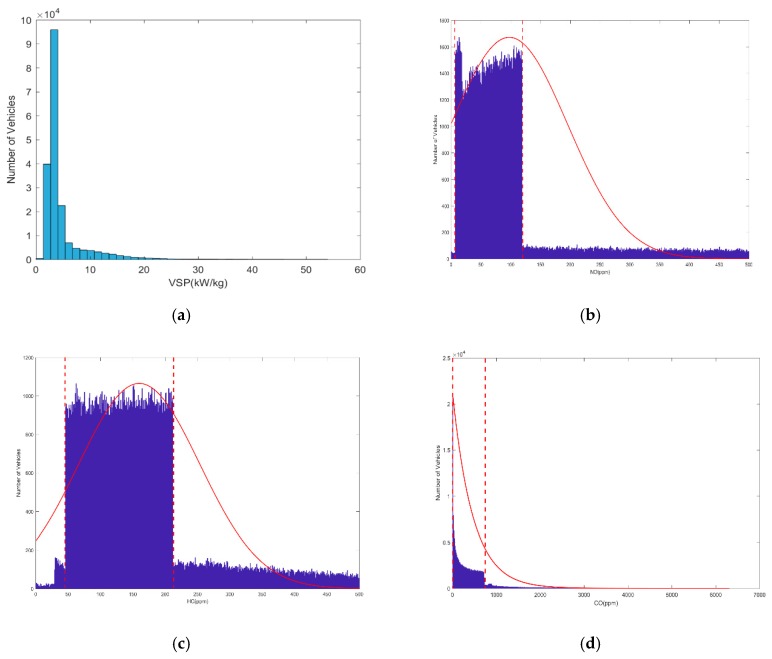
The histogram of vehicle specific power (VSP) and three main types of emissions collected in 192,097 datasets: (**a**) VSP; (**b**) NO; (**c**) HC; (**d**) CO.

The 192,097 emission datasets, which were collected by the IPC in the RSS, include the percentage concentration of $CO,CO_{2},HC,$ and NO, as well as the vehicle speed, acceleration, and the gradient. Since the detection of on-road high-emitting vehicles is related to the percentage concentration of CO,HC,NO and vehicle specific power (VSP), three-dimensional and histogram statistics were adopted to analyze the relationships between these four parameters, as shown in Figure 2 and Figure 3. VSP is calculated by the IPCs by the follow Equation [33],

(2) $$VSP=v\times [a\times (1.1+9.8\times \theta )+0.132]+0.000302\times v^{3}$$

where v is vehicle speed, a is vehicle acceleration and θ is the gradient.

Analysis of the data distribution in Figure 2 and Figure 3, reveals several emission relationships:

1. Few points fall into the zone in which the concentrations of all three emission gases are very high, as shown in Figure 2.
2. According to the U.S National Environmental Protection Agency (EPA), remote sensing data are valid for VSP ranges of 0–20 kW/t [34]; otherwise, the concentrations of CO and HC are likely to have abnormally high values. Figure 3a shows the VSP values in our remote sensing datasets are mostly within the valid range, and the data out of this range were eliminated and deemed invalid.
3. The probability density function that fits the emission datasets is represented by the solid red line in Figure 3b–d. This fit indicates that the NO and HC emission data do not follow a normal distribution, while the CO emission data approximately fit an exponential distribution.
4. Most of the emission datasets are located in a concentration zone, that is marked between two boundaries denoted by the red dashed lines in Figure 3b–d. At both ends of this concentration zone, the number of vehicles has a very significant downward trend.

The purpose of our data analysis is to identify the relationships in the emission data collected by the RSS, so that we can improve the method, and quickly and adaptively recognize high-emitting vehicles.

### 2.3. Data Quality Consideration

To ensure real-time detection of a high emitting vehicle has been performed correctly, the assessment of data quality is based on a comprehensive reference to EPA [34], Hong Kong Transient Emission Test (HKTET) [35], and local standards in Anhui Prov, China, including the following:

1. Monitoring interval: The interval between each vehicle passing the RSS is not less than 1 s, and the monitoring results of the two vehicles passing the RSS time less than 1 s are regarded as invalid.
2. Environmental conditions: The wind speed of the monitoring site shall not exceed 5 m/s; the ambient temperature of the monitoring site shall be in the range of 0–45°; and the relative humidity of the monitoring site shall be less than 80%.
3. Vehicle condition: The VSP, speed and acceleration of the monitored vehicle must be in the range of 0–20 kW/t, 0–90 km/s, and −5~3 km/s/h respectively.
4. $CO_{2}$ concentration: The $CO_{2}$ concentration of monitored vehicle should be maintained at 12–16%.

If any of the above conditions are not met, the corresponding monitoring data in our RSS is considered invalid.

## 3. Methods

**Figure 4:**
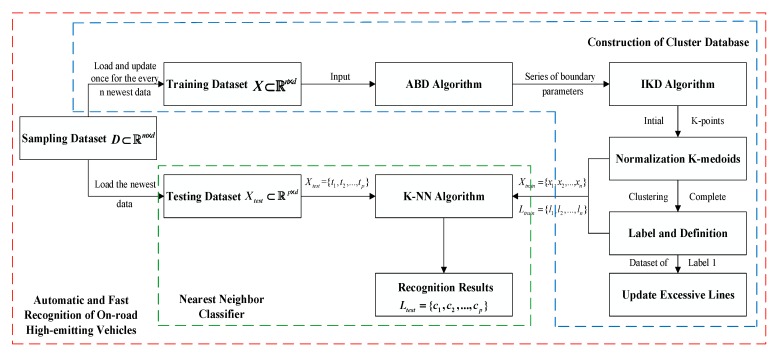
The architecture of the method for the automatic and fast recognition of on-road high-emitting vehicles.

This paper proposes an automatic and fast recognition method that detects on-road high-emitting vehicles, by using the above emission relationships. The proposed method is described in Figure 4. The training dataset $X\subset R^{n\times d}$ is loaded and updated for every *n* new data from the sampling dataset $D\subset R^{m\times d}$, using the automatic boundary detection (ABD) and initial K-center determination (IKD) methods, in order to determine the initial positions of the K-points. After that, the training dataset is normalized to maintain the same weights of different emission gases and clustered by K-medoids. Then, different clusters are labeled and defined. Also, the dataset, label “1”, is extracted to update the cut-points between high-emitting and low-emitting zones of different emission gases. The above processes construct the cluster database in our method, and the outputs, $X_{train}$ and $L_{train}$, are inputs to the nearest-neighbor classifier to complete automatic and fast recognition of the testing dataset. The specific sub-algorithms are described in the next subsection.

### 3.1. Automatic Boundary Detection

Firstly, automatic boundary detection (ABD) is proposed in this paper, in order to improve the adaptability of the high-emitting recognition algorithm. ABD is detailed in Algorithm 1. It loads the most recent n datasets into the database of the IPC. The choice of the n value, and tests to optimize the clustering speed, are discussed in the experimental section.

Because the concentrations of NO, HC, and CO emissions are the focus of this paper, the characteristic dimension of the datasets is 3. Our method is also suitable for datasets with high feature dimensions owing to the advantages of partition-based clustering. In Algorithm 1, the ceil(x), max(X), and histogram(X,δ) call library functions that round up the value x, take the maximum of the array X, and calculate the histogram of the array X and divide it into δ equal intervals, respectively.

**Algorithm 1.** ABD Algorithm**Input:**$D=\{x_{1},x_{2},\ldots ,x_{m}\}$: m 3-dimensional emission datasets; $x_{i1},x_{i2},x_{i3}$: the concentrations of NO, HC, COn: the number of datasets that can be loaded in the main memory**Output:**$\left\{\delta_{1},\delta_{2},\delta_{3}\right\}$: the max concentration of NO, HC, CO;$\left\{X_{max1},X_{max2},X_{max3}\right\}$: the upper boundary values of NO, HC, CO;$\left\{X_{min1},X_{min2},X_{min3}\right\}$: the lower boundary values of NO, HC, CO; 1: load $X=\{x_{m-n+1},x_{m-n+2},\ldots ,x_{m}\}$ from $D=\{x_{1},x_{2},\ldots ,x_{m}\}$2: **for**
j= 1 to 3 **do**3:    **for**
i=1 to *n*
**do**4:      $\delta_{j}=\;ceil(max(X_{ij}))$5:      $Y_{j}=histogram(X_{ij},\delta_{j})$6:    **end for**7:    **for**
i= 1 to $\delta_{j}-1$
**do**8:      $Z_{ij}=Y_{ij}-Y_{(i+1)j}$9:    **end for**10:   **if**
j≤2, **then**11:      $X_{max(j)}=\underset{1\leq i\leq \delta_{j}-1}{argmax}Z_{ij}(X_{ij})$12:      $X_{min(j)}=\underset{1\leq i\leq \delta_{j}-1}{argmin}Z_{ij}(X_{ij})$13:   **else**14:      $X_{max(j)}=\underset{100\leq i\leq \delta_{j}-1}{argmax}Z_{ij}(X_{ij})$15:      $X_{min(j)}=0$16:   **end if**17: **end for**

Figure 3b–d show an example of the results computed by Algorithm 1, with *n* representing the maximum number of samples. The automatic detection boundaries are indicated by the red dotted lines.

### 3.2. Initial K-Center Determination

After the maximum and boundary concentrations of each emission gas are established, the proposed method applies the initial K-center determination algorithm, which is detailed in i Algorithm 2.

The IKD algorithm first calculates the center values of the high- and low-emission zones of each gas, and then it forms matrix A, which contains all the center values. At the end of IKD, the function bitget(i,1:3) is adopted to return a binary value of i from low to high, to automatically generate the initial k center points. Since the ABD and IKD methods are continuous calculation processes, we combined them into a single process termed automatic detection of initial k-center (ADIK).

**Algorithm 2.** IKD Algorithm**Input:**$\delta_{1},\delta_{2},\delta_{3}$: the max concentration of NO, HC, CO$X_{max1},X_{max2},X_{max3}$: the upper-boundary values of NO, HC, CO$X_{min1},X_{min2},X_{min3}$: the lower-boundary values of NO, HC, CO.**Output:**$K=\{u_{1},u_{2},\ldots ,u_{k}\}$: k 3-dimensional initial points1: **for**
j= 1 to 3 **do**2:   $X_{low(j)}=\frac{X_{max(j)}+X_{min(j)}}{2}$3:   $X_{high(j)}=\frac{X_{max(j)}+\delta_{j}}{2}$4: **end for**5: **define** matrix $A\;=\;\left[\begin{array}{ccc} X_{low1} & X_{low2} & X_{low3}\\ X_{high1} & X_{high2} & X_{high3} \end{array}\right]$6: **for**
i= 1 to k
**do**7:   ε=bitget(i−1,1:3)+18:   $u_{i}=(A(\varepsilon (1),1),A(\varepsilon (2),2),A(\varepsilon (3),3))$9: **end for**

### 3.3. Normalization K-Medoids

By running the ADIK algorithms, we acquire the initial positions of k center points. To maintain the same weighting of the NO, HC, and CO emission data, a normalization method is adopted as follows,

(3) $$X_{norm(ij)}=\frac{\delta_{norm}}{\delta_{j}}X_{ij}$$

(4) $$K_{norm(oj)}=\frac{\delta_{norm}}{\delta_{j}}K_{oj}$$

where i=1,2,…,n, j=1,2,3, and o=1,2,…,k.

Then K-medoids are used to cluster the emission datasets, as described in this subsection. The difference between K-means and K-medoids is that the central point $u_{k}$ is selected in different ways,

(5) $$u_{k-means}=\frac{1}{N_{k}}\sum_{x_{i}\in D_{k}}x_{i}$$

(6) $$u_{k-mediods}=\underset{x_{i}\in D_{k}}{argmin}\sum_{x_{j}\in D_{k}}{\left\|x_{j}-x_{i}\right\|}_{2}$$

where $D_{k}$ is the dataset of class k. Compared with K-means, the advantage of using K-medoids to select the central point is that it can effectively eliminate the influence of outliers on the clustering results, and it also increases the total running time of the algorithm. The detailed calculation process of K-medoids is shown in Algorithm 3.

The function repmat(A,n,m) returns an array containing n×m copies of A in the row and column dimensions. The running time of Algorithm 3 largely depends on the size of the clustering datasets and the initial positions of the k center points, which are shown in the experimental section.

**Algorithm 3.** K-Medoids algorithm**Input:**$X_{norm}=\{x_{1},x_{2},\ldots ,x_{n}\}$: n 3D normalized emission datasets extracted from the database D$K_{norm}=\{u_{1},u_{2},\ldots ,u_{k}\}$: k normalized initial pointsε**-** convergence threshold**Output:**$K^{\prime }=\{u_{1}^{\prime },u_{2}^{\prime },\ldots ,u_{k}^{\prime }\}$: k 3-dimensional final K points$B=\{b_{1},b_{2},\ldots ,b_{n}\}$—indicates the class to which $x_{n}$ belongs;Iter**:** iterations of algorithm1: **for**
Iter= 1 to 100 **do**2:   **for**
i= 1 to n **do**3:      $dist={\left\|repmat(X_{norm}(:,i),1,k)-K_{norm}\right\|}_{2}$4:      [~,index]=min(dist)5:      B(i)=index6:   **end for**7:   **for**
i=1 to k
**do**8:      $X=X_{norm}(:,find(B==i))$9:      N=size(X,2)10:      **for**
j=1 to N
**do**11:         $totaldist(j)=sum({\left\|X-X(:,j)*ones(1,N)\right\|}_{2})$12:      **end for**13:      [~,mindex]=min(totaldist)14:      $K^{\prime }(:,i)=X(:,minindex)$15:   **end for**16:   **if**
$\left\|K^{\prime }-K_{norm}\right\|\leq \varepsilon$17:      **break**18:   **end if**19:  $K_{norm}=K^{\prime }$20: **end for**

### 3.4. Label and Definition

After clustering is finished, different clusters of emission datasets can be labeled by the formula,

(7) $$Label=B\times {\left(1:k\right)}^{T}$$

where $B=\{b_{1k},b_{2k},\ldots ,b_{n}{}_{k}|b_{n}{}_{k}\in \{0,1\}\}$ is as described in the above subsection.

The unlabeled samples in the training datasets are transformed into labeled samples by this method. The labels and definitions of the results are shown in Table 1.

### 3.5. Nearest Neighbor Classifier

Once the clustered datasets have been established and labeled, the K-NN algorithm, which is shown in Algorithm 4, is applied to rapidly detect high-emitting vehicles.

**Algorithm 4.** K-NN algorithm**Input:**$X_{train}=X_{norm}=\{x_{1},x_{2},\ldots ,x_{n}\}$—n 3-dimensional training emission datasets$X_{test}=\{t_{1},t_{2},\ldots ,t_{p}\}$—m 3-dimensional testing emission datasets$L_{train}=\{l_{1},l_{2},\ldots ,l_{n}\}$—the labels of training emission datasetsk—initial parameters of K-NN**Output:**$L_{test}=\{c_{1},c_{2},\ldots ,c_{p}\}$—the labels of testing emission datasets1: **for**
i= 1 to p
**do**2:   $diff=repmat(X_{test}(i),[n,1])-X_{train}$3:   $dist=\sqrt{\sum_{j=1}^{3}diff{\left(j\right)}^{2}}$4:   $\left[X_{sort},IX]=sort(dist\right)$5:   $totallab=L_{train}(IX(1:k))$6:   $L_{test}(i)=mode(totallab)$7: **end for**

K-NN calculates the Euclidean distance between the testing sample and all training samples, and then the k training samples, closest to the test sample, are selected. The value that appears most frequently in the labels, corresponding to k training samples, is regarded as the label of the testing sample.

### 3.6. Update Cut-Points of Excessive Emissions

As the dataset labeled “1” is defined as a “No Excessive Emissions” zone, it can be extracted to update the cut-points that define high-emitting and low-emitting zones. In the approach proposed in this paper, the maximum concentrations of different emissions gases, which are regarded as the cut-points, are calculated in the dataset labeled “1”, and they are updated for every *n* newest input dataset.

## 4. Experiments and Discussion

In order to verify the advantages of the proposed method, we performed several experiments, which are described in this section. All experiments were conducted on a Windows10-64bit operation system with an Inter I5-7300U 2.71 Hz CPU and 8 GB RAM.

### 4.1. Experiment to Compare Clustering Methods

**Figure 5:**
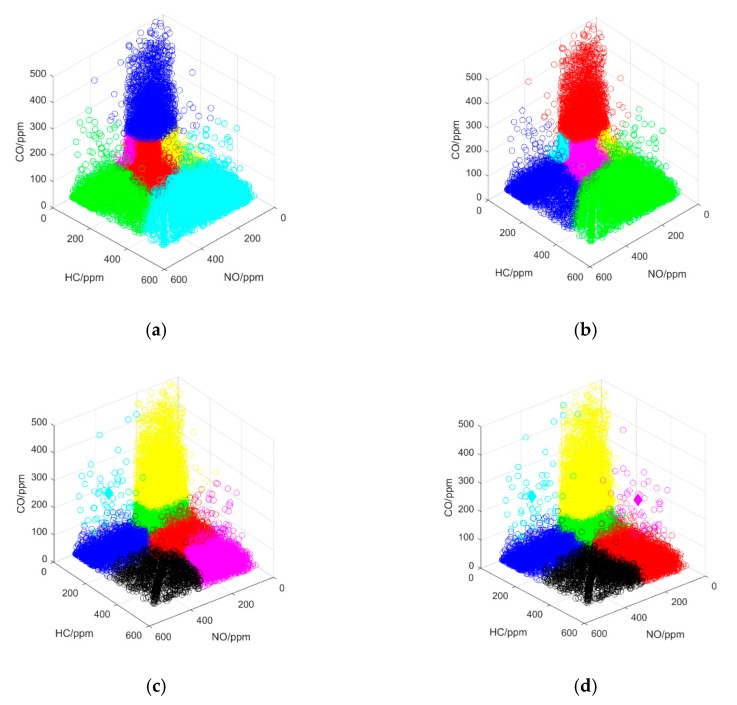
The results of experiments to compare clustering methods: (**a**) K-means; (**b**) K-medoids; (**c**) ADIK+K-means; (**d**) proposed method.

The performance of our proposed method was tested in the first experiment, which entailed the qualitative and quantitative analyses to compare K-means, K-medoids, and ADIK+K-means. All clustering processes were performed 30 times, and the average results are reported in Table 2. The clustering process, with the smallest total squared distance, was used as the sample for the qualitative analysis, which is shown in Figure 5 (emissions data were normalized).

By comparing the clustering results in Figure 5a–d, we found that our method effectively solved the problem of selecting the initial center of clustering, and the 60,000 datasets were divided into our defined emission zones. The outliers that influence K-means were eliminated by K-medoids, as shown in Figure 5c,d, and the proposed method obtained the best clustering results of the four tested methods.

Then the effectiveness of the clustering algorithms was tested using three types of qualitative indicators: The running time of the algorithm (TIME), the Davies Bouldin Index (DBI), and the Dunn Validity Index (DVI) [36,37],

(8) $$DBI=\frac{1}{k}\sum_{i=1}^{k}\underset{j\neq i}{max}(\frac{avg(C_{i})+avg(C_{j})}{d_{cen}(u_{i},u_{j})})$$

(9) $$DVI=\underset{1\leq i\leq k}{min}\left\{\underset{j\neq i}{min}(\frac{d_{min}(C_{i},C_{j})}{\underset{1\leq l\leq k}{max}diam(C_{l})})\right\}$$

in which:

(10) $$avg(C)=\frac{2}{\left|C\right|(\left|C\right|-1)}\sum_{1\leq i<j\leq \left|C\right|}dist(x_{i},x_{j})$$

(11) $$d_{cen}(C_{i},C_{j})=dist(u_{i},u_{j})$$

(12) $$d_{min}(C_{i},C_{j})=min_{x_{i}\in C_{i},x_{j}\in C_{j}}dist(x_{i},x_{j})$$

(13) $$diam(C)=max_{1\leq i<j\leq \left|C\right|}dist(x_{i},x_{j})$$

where avg(C) is the mean distance between samples in cluster C; $d_{cen}(C_{i},C_{j})$ is the distance between the center points of cluster $C_{i}$ and $C_{j}$; $u=\frac{1}{\left|C\right|}\sum_{1\leq i\leq \left|C\right|}x_{i}$, which is the center point of C; $d_{min}(C_{i},C_{j})$ is the distance between the nearest samples of clusters, $C_{i}$ and $C_{j}$; and diam(C) is the longest distance between samples in cluster C.

The smaller the TIME value, the higher the efficiency of the algorithm; the smaller the DBI and the larger the DVI, the better the clustering performance. As shown in Table 2, the ADIK method was adopted to rapidly determine the initial K-center, which was able to effectively reduce the convergence speed of the clustering method, reduce the DBI, and increase the DVI. The K-medoids approach eliminated the influence of outliers, and its DBI and DVI were better than those of the K-means method.

For the next step, the size of the clustering dataset and the clustering time were comprehensively considered. We chose n = 10,000 as the newest input training dataset. This dataset sizes not only ensured that the data characteristics were retained, but also allowed real-time updates of the RSS data. The average running time was less than 5 s, which satisfied the requirements for adaptability and real-time performance.

### 4.2. Performance Evaluation of the Nearest-Neighbor Classifier

After the clustering emission database was established, the performance of our classifier was tested. The qualitative and quantitative analytical methods, from experiment A, were adopted for this experiment as well.

**Figure 6:**
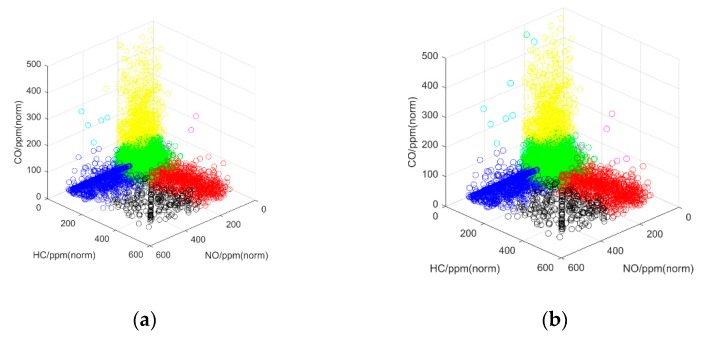
The comparison results of clustering experiments: (**a**) testing sets; (**b**) validation sets.

The most recent 10,000 emission datasets, collected by the RSS were used as the training sets, and the training labels were the emission recognition results of our clustering database. The testing sets were accumulated by monitoring the emission dataset of each new vehicle that passed through the RSS, and the recognition results of 10,000 testing sets were compared with the validation sets, obtained by the clustering algorithm in the experiment, as shown in Figure 6.

The results of the quantitative analysis in Figure 6 show that our classifier obtained a better recognition result. Then, Precision (PRE) and Recall (REC) were used to test the performance of our classifier (Table 3). The formulas for these two indicators are,

(14) $$PRE=\frac{TP}{TP+FP}$$

(15) $$REC=\frac{TP}{TP+FN}$$

where TP, FP, and FN denote true positive, false positive, and false negative, respectively.

**Figure 7:**
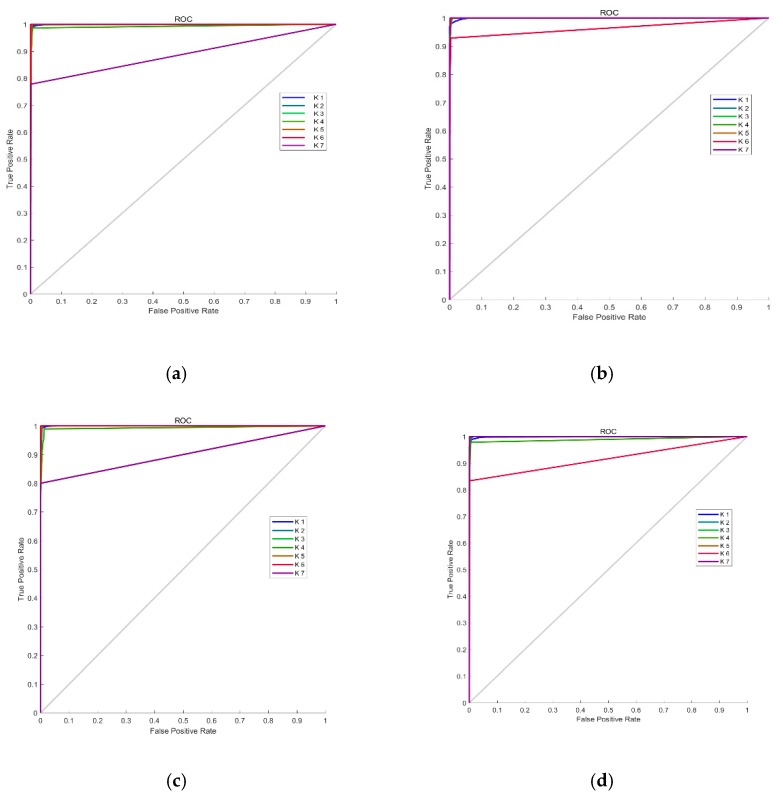
The receiver operator characteristic (ROC) of different testing datasets: (**a**) dataset of Day 1; (**b**) dataset of Day 2; (**c**) dataset of Day 3; (**d**) dataset of Day 4.

Because the number of categories in our classified samples was unbalanced, the true positive rate (TPR) and false positive rate (FPR) were critical performance indicators. Therefore, the receiver operator characteristic (ROC) [38] based on these two indicators was adopted as shown in Figure 7.

Then, the area under the curve (AUC) [39] was calculated to test the final performance of the classifier, and the results are shown in Table 3. By calculating the various performance indexes for the four sample datasets collected at different times and places, we found that our classifier achieved good results. Here, we paid more attention to the evaluation indicators for category, $k_{1}$ because $k_{1}$ represents vehicles that do not exceed the standard, while all other categories represent vehicles that exceed the standard. The results of this quantitative experiment show that our classifier could accurately recognize the non-exceeding category, $k_{1}$ and the exceeding categories $k_{2}\sim k_{5}$, and it achieved an adequate recognition rate for the emission-exceeding categories, $k_{6}$ and $k_{7}$. The reason for this difference in classification performance might be the small sample size for $k_{6}$ and $k_{7}$. Additionally, the results of tens of thousands of experiments show that the average recognition speed of our classifier was less than 0.1 s per detected vehicle, which meets the requirements for fast and automatic recognition.

When a new vehicle passes through the RSS, the classifier in the system will automatically distribute the detection result of the new vehicle into a category, according to the trained model, and the LED display will rapidly display the detection results. At the same time, the system would add count information to the database of monitoring results, and the information index is the license plate number of the new recognized car. For example, if a new car were to be assigned to category $k_{4}$, then the counts of excessive NO and HC emissions will increase once they are added to the database of detection results. If the total counts of this car exceed the limit, the system will blacklist the license plate number of this car and upload its information to inform law enforcement authorities.

The advantage of this processing method is that it eliminates some of the factors that might affect a single instantaneous monitoring system. The potential effects might include, noise from the optical equipment and the external environment and sudden acceleration or deceleration of a vehicle.

### 4.3. The Experiment for Detection Vehicles Exceeding the Standard Rate

In the experiment reported in this sub-section, the automatic and fast recognition method for detecting on-road high-emitting vehicles was tested for cases, in which the standard rate was exceeded. Six experimental datasets, obtained from two different geographical locations, Shijiazhuang and Hefei, were collected by the RSSs at different times, and each dataset contained 10,000 telemetric data points. The results of the experiment are shown in Table 4, which shows that the average rates of standards being exceeded and not exceeded were 27.69% and 72.31%, and the average rates of excessive NO, HC, and CO emissions were 10.53%, 12.98%, and 7.03% respectively.

### 4.4. The Experiment for Self-Adaptive Cut-Points

Experimental datasets were collected from three different geographical locations, which had been described in Section 2.1, for three days. As the cut-off points in the system were updated every 10,000 new datasets, we took the average of the cut-points in a day. The experimental results for self-adaptive cut-off points are shown in Table 5. We can find that the cut-off points in the table change with time and location, which proves that our proposed method has good adaptability.

It can be seen from the results in Table 5 that the cut-off points in our system do not change much with time, but with the change in geographical locations, a more obvious change takes place. As this experiment was done only verify to the adaptability of our proposed method, the relationships between cut-points and time, locations, outside environment, and different equipment need to be evaluated with more experimental datasets, which will be further demonstrated in future research work.

## 5. Conclusions

This paper proposes a method for the automatic and fast recognition of on-road high-emitting vehicles, called AFR-OHV. The first step in the AFR-OHV method is to adaptively determine the initial clustering center, according to the distribution characteristics of the most recently input RSS datasets, and to counteract the effects of environmental change to some extent. The second step in AFR-OHV is the normalization of the K-medoids clustering of the RSS datasets. After that, the RSS datasets are labeled and divided into different defined emission zones to construct a clustering database, and then the cut-points are updated automatically. The last step is to recognize high-emitting vehicles, which pass through RSS by a nearest-neighbor classifier, and to update the clustering database.

As reported in the experimental section, the performance of the method was verified using real data collected by RSS from December 2018 to January 2019 on Xueyuan Road, Shijiazhuang City, Hopei Prov, China, and Yangqiao Road, Hefei City, Anhui Prov, China. Different clustering methods were selected for comparison, and the experimental results show that the running time, DBI, and DVI resulting from our method were superior to those obtained using three other methods, namely, ADIK + K-means, K-medoids and K-means. Our classifier also had better performance indexes, i.e., PRE, REC, and AUC. In the last step, the rates of exceeded standards were calculated using multiple emission datasets collected by the RSS in two different geographical locations. The calculated rates provide reference values for law enforcement departments to establish evaluation criteria for on-road high-emitting vehicles detected by remote sensing systems.

The limitation of this paper’s work is that, when optical remote sensing systems, that are developed by different research institutions or companies, are used to detect on-road high-emitting vehicles, the distribution of the emission datasets might be significantly different. In our future work, we will research transfer learning and meta learning in an aim to improve our learning method. The objective is to improve the model so that it can be effectively applied to other optical remote sensing systems after training with a dataset from one set of optical remote sensing systems. In addition, we will research multi-RSS networking on adjacent streets to further reduce the monitoring error and improve the recognition accuracy.

## Figures and Tables

**Table 1:** The labels and definitions of different k categories.

$k_{i}$	NO		HC		CO		Definition
	High	Low	High	Low	High	Low	
$k_{1}$	0	0	0	0	0	0	No Excessive Emissions
$k_{2}$	1	0	0	0	0	0	Excessive NO
$k_{3}$	0	0	1	0	0	0	Excessive HC
$k_{4}$	1	0	1	0	0	0	Excessive NO and HC
$k_{5}$	0	0	0	0	1	0	Excessive CO
$k_{6}$	1	0	0	0	1	0	Excessive NO and CO
$k_{7}$	0	0	1	0	1	0	Excessive HC and CO
$k_{8}$	1	0	1	0	1	0	Excessive NO, HC, and CO

**Table 2:** The performance test of different clustering algorithms.

Emission Dataset	Proposed Algorithm			ADIK + K-Means			K-Medoids			K-Means		
Magnitude	Time (s)	DBI	DVI	Time (s)	DBI	DVI	Time (s)	DBI	DVI	Time (s)	DBI	DVI
5000	2.62 ± 0.44	19.68 ± 3.14	0.0102 ± 0.0013	2.16 ± 0.51	27.05 ± 2.32	0.0054 ± 0.0009	2.97 ± 1.54	17.82 ± 9.84	0.0039 ± 0.0014	2.73 ± 1.54	21.84 ± 13.37	0.0028 ± 0.0009
8000	4.47± 0.40	29.51 ± 4.04	0.0027 ± 0.0005	2.50 ± 0.33	42.83 ± 3.47	0.0028 ± 0.0008	3.79 ± 1.78	37.68 ± 14.55	0.0028 ± 0.0004	3.09 ± 1.31	52.57 ± 17.38	0.0019 ± 0.0003
10,000	4.68 ± 1.39	32.96 ± 4.24	0.0045 ± 0.0011	2.66 ± 0.31	44.51 ± 4.26	0.0028 ± 0.0010	5.39 ± 1.93	44.38 ± 16.79	0.0031 ± 0.0006	4.07 ± 1.71	55.84 ± 20.77	0.0017 ± 0.0005
20,000	15.83 ± 2.45	34.30 ± 3.25	0.0025 ± 0.0006	3.30 ± 0.74	48.18 ± 4.96	0.0030 ± 0.0009	17.54 ± 3.18	52.94 ± 23.18	0.0039 ± 0.0007	5.67 ± 1.21	61.29 ± 21.23	0.0028 ± 0.0008
30,000	45.36 ± 7.32	36.95 ± 3.71	0.0028 ± 0.0009	3.93 ± 0.95	53.49 ± 5.84	0.0018 ± 0.0005	51.28 ± 6.49	60.17 ± 20.62	0.0027 ± 0.0005	6.29 ± 1.57	67.40 ± 19.83	0.0018 ± 0.0004
40,000	91.60 ± 12.03	41.85 ± 4.73	0.0029 ± 0.0007	5.49 ± 1.47	56.81 ± 5.07	0.0023 ± 0.0005	107.45 ± 10.39	64.73 ± 24.25	0.0022 ± 0.0004	6.98 ± 1.81	70.72 ± 18.46	0.0016 ± 0.0003
50,000	110.36 ± 19.88	49.61 ± 4.52	0.0038 ± 0.0010	7.41 ± 1.83	60.03 ± 5.92	0.0027 ± 0.0007	125.81 ± 14.84	68.35 ± 23.49	0.0028 ± 0.0006	8.47 ± 2.03	76.93 ± 20.32	0.0019 ± 0.0003

**Table 3:** The performance test results of our classifier.

Testing Dataset	Dataset of Day I			Dataset of Day 2			Dataset of Day 3			Dataset of Day 4		
Categories	PRE	REC	AUC	PRE	REC	AUC	PRE	REC	AUC	PRE	REC	AUC
$k_{1}$	0.9980	0.9898	0.9929	0.9820	0.9979	0.9740	0.9994	0.9857	0.9919	0.9983	0.9816	0.9888
$k_{2}$	0.9802	0.9682	0.9840	0.9688	0.9848	0.9914	0.9327	0.9945	0.9950	0.8982	0.9862	0.9861
$k_{3}$	0.9688	0.9963	0.9360	0.9914	0.9851	0.9911	0.9430	0.9991	0.9852	0.9667	0.9937	0.9959
$k_{4}$	0.9368	0.8750	0.9962	0.9395	0.9983	0.9994	0.9861	0.7634	0.9917	0.9707	0.7133	0.8740
$k_{5}$	0.8965	0.9982	0.9958	0.9884	0.9440	0.9916	0.9088	0.9966	0.9964	0.8504	1.0000	0.9942
$k_{6}$	1.0000	0.6667	0.9916	1.0000	0.7476	0.9457	1.0000	0.1667	0.9935	1.0000	0.6666	0.8868
$k_{7}$	1.0000	0.5556	0.8837	0.9800	0.7147	0.9983	1.0000	0.6000	0.8536	1.0000	0.4000	0.9980

**Table 4:** The results of the experiment for detecting the rate of exceeded emissions.

	Datasets	Loc. I 1	Loc. I 2	Loc. I 3	Loc. II 1	Loc. II 2	Loc. II 3	Avg
Categories								
Excessive NO		7.92%	8.17%	7.25%	7.25%	7.87%	8.26%	7.79%
Excessive HC		10.37%	10.90%	9.39%	11.09%	8.94%	10.83%	10.25%
Excessive CO		7.70%	5.64%	8.07%	5.80%	7.50%	6.29%	6.83%
Excessive NO and HC		2.64%	2.88%	2.33%	2.79%	2.65%	2.47%	2.63%
Excessive NO and CO		0.15%	0.06%	0.14%	0.06%	0.11%	0.07%	0.10%
Excessive HC and CO		0.11%	0.08%	0.11%	0.05%	0.07%	0.12%	0.09%
Excessive NO, HC, and CO		0.00%	0.01%	0.00%	0.00%	0.02%	0.00%	0.01%
Excessive		28.89%	27.74%	27.29%	27.04%	27.16%	28.04%	27.69%
No Excessive		71.11%	72.26%	72.71%	72.96%	72.84%	71.96%	72.31%

**Table 5:** The performance test results of our classifier.

	Cut-Points	Dataset of Day I			Dataset of Day 2			Dataset of Day 3		
Locations		CO	HC	NO	CO	HC	NO	CO	HC	NO
Shijiazhuang, Hebei		1.2047%	240 ppm	203 ppm	1.2549%	246 ppm	205 ppm	1.2273%	242 ppm	202 ppm
Hefei, Anhui		1.5472%	258 ppm	222 ppm	1.5194%	253 ppm	215 ppm	1.5249%	255 ppm	220 ppm
Zibo, Shandong		1.1122%	211 ppm	193 ppm	1.2371%	216 ppm	190 ppm	1.1844%	214 ppm	193 ppm
